# Multi-Omics Analysis of GNL3L Expression, Prognosis, and Immune Value in Pan-Cancer

**DOI:** 10.3390/cancers14194595

**Published:** 2022-09-22

**Authors:** Pei Liu, Wenjia Guo, Ying Su, Chen Chen, Yuhua Ma, Ping Ma, Cheng Chen, Xiaoyi Lv

**Affiliations:** 1 College of Information Science, Engineering Xinjiang University, Urumqi 830046, China; 2 College of Software, Xinjiang University, Urumqi 830046, China; 3 Xinjiang Medical University Affiliated Tumor Hospital, Urumqi 830011, China; 4 Xinjiang Cloud Computing Application Laboratory, Karamay 834099, China; 5 Karamay Central Hospital, Karamay 834099, China

**Keywords:** GNL3L, pan-cancer, multi-omics, prognostic analysis, immune

## Abstract

**Simple Summary:**

Guanine nucleotide-binding protein-like 3-like (GNL3L) is a novel GTP-binding nucleolar protein. In this study, we analyzed the expression, prognosis, and immune roles of GNL3L in pan-cancer from multiple omics analyses. The final results showed that GNL3L is differentially expressed in a variety of cancers, plays a prognostic role, and has good immune value. Moreover, GNL3L may affect the occurrence of cancer through processes such as ribonucleoprotein, ribosomal RNA processing, and cell proliferation. At the same time, we established an esophageal cancer (ESCA) prediction model with strong predictive ability and proved that GNL3L can significantly affect the proliferation ability of esophageal cancer cells through clone formation assays. In conclusion, GNL3L is an important biomarker.

**Abstract:**

Guanine nucleotide-binding protein-like 3-like protein (GNL3L) is a novel, evolutionarily conserved, GTP-binding nucleolar protein. This study aimed to investigate the expression, prognosis, and immune value of GNL3L in pan-cancer from multiple omics analyses. Firstly, the expression and prognostic value of GNL3L in pan-cancer were discussed using the TIMER2 database, the GEPIA database, the cBioportal database, COX regression analysis, and enrichment analysis. The association of GNL3L with tumor mutational burden (TMB), tumor microsatellite instability (MSI), mismatch repair (MMR) genes, and immune cells was then analyzed. Finally, an esophageal cancer (ESCA) prediction model was established, and GNL3L clone formation assays were performed. The final results showed that GNL3L is differentially expressed in the vast majority of cancers, is associated with the prognosis of various cancers, and may affect cancer occurrence through processes such as ribonucleoprotein, ribosomal RNA processing, and cell proliferation. At the same time, it was found that the correlation between GNL3L and TMB, MSI, MMR, and various immune cells is significant. The established ESCA prediction model had a strong predictive ability, and GNL3L could significantly affect the proliferation of esophageal cancer cells. In conclusion, GNL3L may serve as an important prognostic biomarker and play an immunomodulatory role in tumors.

## 1. Introduction

For decades, because of the high mortality rate, cancer has brought a serious burden to the world [1]. Research related to cancer has been the focus of scientific studies, and related researchers are constantly studying cancer from a multi-omics perspective such as imaging, spectroscopy, and genetics [2]. Cancer has emerged as a major public health issue around the world, and the incidence and mortality of cancer are increasing every year [3]. Therefore, finding biomarker genes that can help is an important means of preventing and treating cancer. However, there are problems, such as small study sample sizes, low statistical efficiency, and poor reproducibility in the genetic study of a single cancer, and some genes play a role in multiple cancers. Therefore, gene expression, prognosis, and immune analysis in pan-cancer are very important [4]. Pan-cancer analysis research relates to the simultaneous analysis of multiple types of cancers to find the common features of these cancers and provide broad-spectrum targets for clinical diagnosis and the treatment of multiple cancers. GNL3L is a newly discovered GTP-binding nucleolar protein that can regulate the mitotic cycle of eukaryotic cells and affect cell proliferation and apoptosis [5,6]. Through the binding and regulation experiments of TRFI and GNL3L, Tsai et al. found that GNL3L can bind to telomerase repeat-binding factor 1 (TRF1), stabilize TRF1 during mitosis, and promote the transition of cells from metaphase to anaphase [7,8]. Later, Tsai and Meng et al. found that GNL3L acts as a potential pro-oncogenic factor by blocking ubiquitination to stabilize mouse double microsomes 2 (MDM2), thereby having an inhibitory effect on p53 function, and demonstrated that the knockdown of GNL3L promotes G2/M phase block and upregulates p53 downstream targets [9]. Furthermore, TI Jose et al. demonstrated that GNL3L is a nuclear–cytoplasmic transporter that promotes cell S-phase progression [8]. Later, Thoompumkal et al. found that GNL3L upregulates NF-κB-dependent transcriptional activity by regulating the expression of the p65 subunit of NF-κB, thereby knocking down p65 to disrupt the anti-apoptotic function of GNL3L, demonstrating that GNL3L plays a role in tumorigenesis and progression by regulating NF-κB signaling [10]. According to research, GNL3L plays a significant role in many cancers, such as renal cancer, colorectal cancer, esophageal cancer, liver and rectal cancer, and breast cancer [8,10,11]. These aforementioned studies suggest that GNL3L may be a prognostic and therapeutic biomarker of great significance. However, a comprehensive analysis of GNL3L expression, prognosis, and immunity on the pan-cancer level has not yet emerged.

Therefore, in this study, the method of multi-omics analysis was used to study GNL3L in pan-cancer from multiple perspectives. We first found the expression of GNL3L in 33 cancers in this research, correlation with molecular subtypes and clinical stages in human cancers, genetic variants in human cancers, and copy number alteration (CNA), and we used associated gene enrichment analysis to discuss the prognostic significance of GNL3L. Then, in order to explore its immune value, the correlation of GNL3L with TMB, MSI, MMR, and various immune cells was analyzed and we made an analysis of the immune tumor microenvironment. During the analysis, GNL3L was found to be highly expressed and poorly prognosed in ESCA and an independent factor in ESCA prognosis. Therefore, with the intention of further investigating the prognostic value and clinical importance of GNL3L, ESCA was selected for experimentation and verification, an ESCA prediction model based on GNL3L was established, and clone formation assays of GNL3L overexpression and knockdown were performed. The results of this study indicated that GNL3L is highly expressed in a variety of cancers, has good prognostic and immune value, and is a potential prognostic immune biomarker.

## 2. Materials and Methods

### 2.1. Expression Analysis of the GNL3L Gene

The gene expression of GNL3L among 33 forms of cancer was analyzed in the TIMER2 database (http://timer.cistrome.org/ (accessed on 15 February 2022)) [12]. The GEPIA database (http://gepia2.cancer-pku.cn (accessed on 15 February 2022)) was utilized to evaluate the differential expression of the GNL3L gene for cancer types that were not matched to normal tissues in the TIMER2 database [13]. Meanwhile, protein expression analysis of GNL3L in six cancer tissues was performed using the CPTAC dataset from the UALCAN database (http://ualcan.path.uab.edu/analysis-prot.html (accessed on 15 February 2022)) [14,15]. In all analysis results, * *p* < 0.05; ** *p* < 0.01, and *** *p* < 0.001.

### 2.2. Immunohistochemistry Staining

The HPA (https://www.proteinatlas.org/ (accessed on 15 February 2022)) database is based on proteomic, transcriptomic, and systems biology data, which can map tissues, cells, organs, etc. To continue to assess differences in GNL3L protein expression levels, immunohistochemical images of 20 tumors were studied from the HPA database.

### 2.3. Cancer Immune and Molecular Subtyping

The TISIDB database (http://cis.hku.hk/TISIDB/index.php (accessed on 15 February 2022)) collects a large number of human cancer datasets and can be used in human cancer immune and molecular subtype analysis [16]. Therefore, the TISIDB database was used to investigate the correlation between GNL3L and molecular and immunological subtypes of 33 cancers.

### 2.4. Genetic Variation and CNA Variation Analysis

The genetic variation in the GNL3L gene was analyzed using the cBioPortal database (https://www.cbioportal.org/ (accessed on 15 February 2022)) [17]. At the same time, the cBioPortal database was used to download CNA data for 33 tumors, and R language was used for correlation analysis. R4.1.0 was used for data collation and analysis.

### 2.5. Prognosis Analysis

The Cancer Genome Atlas (TCGA) database was used to download pan-oncogene RNA-seq data and clinical data for 33 malignancies from the Xena Browser (https://xena.ucsc.edu/ (accessed on 15 February 2022)) [18,19]. Univariate OS, PFI, and DSS prognostic analyses were then performed for 33 cancer types using R4.1.0.

### 2.6. Correlation Analysis of Tumor Mutation Burden, Tumor Microsatellite Instability, and Mismatch Repair Gene Expression

TMB refers to the number of DNA mutations carried by a tumor that may lead to the production of neoantigens [20]. MSI refers to the phenomenon of changes in the length of microsatellite sequences due to insertion or deletion mutations during DNA replication [21]. MMR is an important DNA repair mechanism that can accurately identify and repair base mismatches generated during DNA replication or recombination [22]. Somatic mutation data for 33 cancers were downloaded from the TCGA database, and the MAF files were analyzed using the R package “maftools” to calculate tumor TMB and MSI values. MMR detection mainly detects the expression of four proteins, MLH1, PMS2, MSH2, and MSH6, in cancer tissues. The correlation between the GNL3L gene and the expression levels of the four MLH1, PMS2, MSH2, and MSH6 protein genes was analyzed using the TCGA database.

### 2.7. Immune Cell Correlation Analysis

With two different algorithms, the CIBERSORT algorithm calculates the proportion of immunological cells of many sorts in each sample based on LM22, and then performs correlation analysis with GNL3L in each sample [23,24]. The ssGSEA algorithm is an extension of the GSEA method. ssGSEA allows for the definition of an enrichment score that represents the absolute enrichment of gene sets in each sample within a given dataset, then ranks and normalizes the gene expression values of a given sample to finally generate an enrichment score [25]. The raw data of the two algorithms were obtained from the pan-cancer data of the TCGA database, and R4.1.0 was used for data processing and analysis.

### 2.8. Related Gene Enrichment Analysis

The top 100 proteins associated with GNL3L were obtained using the STRING website (https://string-db.org/ (accessed on 15 February 2022)), a protein–protein interaction network (PPI) was constructed, and these protein-interacting networks were represented in the Cytoscape software [26,27]. Meanwhile, the top 100 genes associated with GNL3L expression were obtained using the GEPIA2 database. The Pearson correlation coefficient values of these 100 genes and GNL3L are all between 0.74 and 0.62, and the *p* values are all less than 0.05. The genes corresponding to the 100 proteins in the PPI and the top 100 genes obtained from the GEPIA2 database were intersected, and then a correlation analysis was performed on the intersected genes to draw a chord diagram. Finally, enrichment analysis was performed on all genes in the two datasets obtained above [28,29]. In addition to this, the cellular localization and function of GNL3L were analyzed using the GeneCards database [30].

### 2.9. Establishment of ESCA Prediction Model

Cox regression was utilized to investigate the related characteristics of survival and prognosis in ESCA patients, and the related factors were used to construct a nomogram and establish ESCA prediction models [31]. Finally, the calibration curve and ROC curve were utilized to verify and estimate the predictive accuracy of the nomogram [32].

### 2.10. Cell Culture and Clone Formation Assay

Esophageal cancer cell lines (KYSE30 and KYSE150) were obtained from the Shanghai Cell Bank (Shanghai, China), and all cell lines were supplemented with 10% fetal bovine serum, 100 units/mL penicillin, 100 μg/mL streptomycin, and 5% CO_2_ in DMEM high-glucose medium. For clone formation assays of GNL3L overexpression and knockdown, KYSE30 and KYSE150 cells were seeded into 6-well plates (1000 cells/well). After 14 days of culture, the formation of cell clones was examined, fixed with 4% paraformaldehyde, stained with 0.1% crystal violet, and colonies containing at least 50 cells were counted for analysis.

## 3. Results

### 3.1. GNL3L Expression Analysis

**Figure 1:**
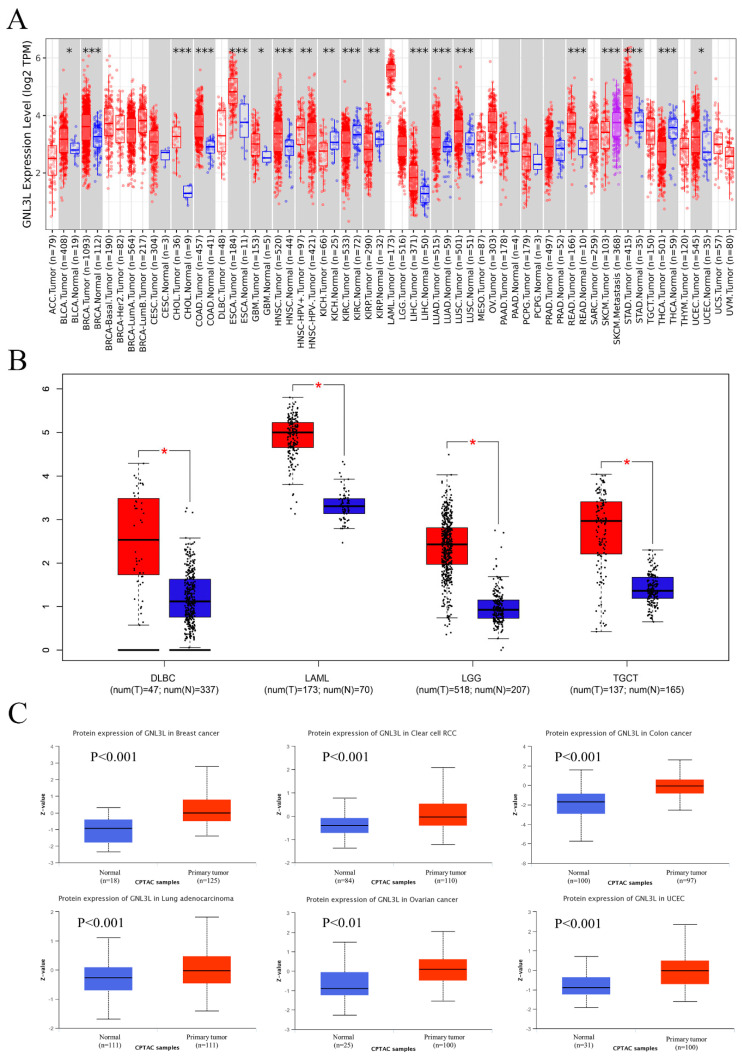
GNL3L gene expression levels and protein expression levels. Red represents patient samples and blue represents normal samples. (**A**) Expression of GNL3L gene in ACC, BLCA, BRCA, BRCA−Basal, BRCA−Her2, BRCA−LumA, CESC, CHOL, COAD, DLBC, ESCA, GBM, HNSC, HNSC−HPV+, HNSC−HPV−, KICH, KIRC, KIRP, LAML, LGG, LIHC, LUAD, LUSC, MESO, OV, PAAD, PCPG, PRAD, READ, SARC, SKCM, SKCM, metastasis, STAD, TGCT, THCA, THYM, UCEC, UCS, and UVM based on TIMER2 database. (**B**) Expression of GNL3L gene in DLBC, LAML, LGG, and TGCT based on GEPIA database. (**C**) Expression of GNL3L protein in breast cancer, clear cell RCC, colon cancer, lung adenocarcinoma, ovarian cancer, and UCEC based on CPTAC database. * *p* < 0.05; ** *p* < 0.01 and *** *p* < 0.001.

The TIMER2 database analysis revealed that GNL3L was immensely expressed in BLCA (bladder urothelial carcinoma), BRCA (breast invasive carcinoma), CHOL (cholangiocarcinoma), COAD (colon adenocarcinoma), ESCA, GBM (glioma multiforme), HNSC (head and neck squamous cell carcinoma), LIHC (hepatocellular G liver cancer), LUAD (lung adenocarcinoma), LUSC (lung squamous cell carcinoma), READ (rectal adenocarcinoma), STAD (gastric cancer), and UCEC (endometrial carcinoma). However, the GNL3L gene was found in KIHC (hepatocellular liver cancer), KIRC (renal clear cell carcinoma), KIRP (renal papillary cell carcinoma), SKCM (skin melanoma), and THCA (thyroid cancer) (Figure 1A).

The GTEx dataset was utilized to assess the differential expression of GNL3L among normal and malignant tissues for malignancies that were not matched as normal tissues in the TIMER2 database. The GEPIA database analysis results show that GNL3L is highly expressed in DLBC (diffuse large B-cell lymphoma), LAML (acute myeloid leukemia), LGG (low-grade glioma of the brain), and TGCT (testicular cancer) tumor tissues (Figure 1B).

Using the UALCAN database for the protein expression analysis of the CPTAC dataset, the total GNL3L protein acquired from the CPTAC dataset showed raised protein expression levels of GNL3L in tissues of breast cancer, clear cell RCC, colon cancer, lung adenocarcinoma, ovarian cancer, and UCEC compared to normal tissues (Figure 1C).

**Figure 2:**
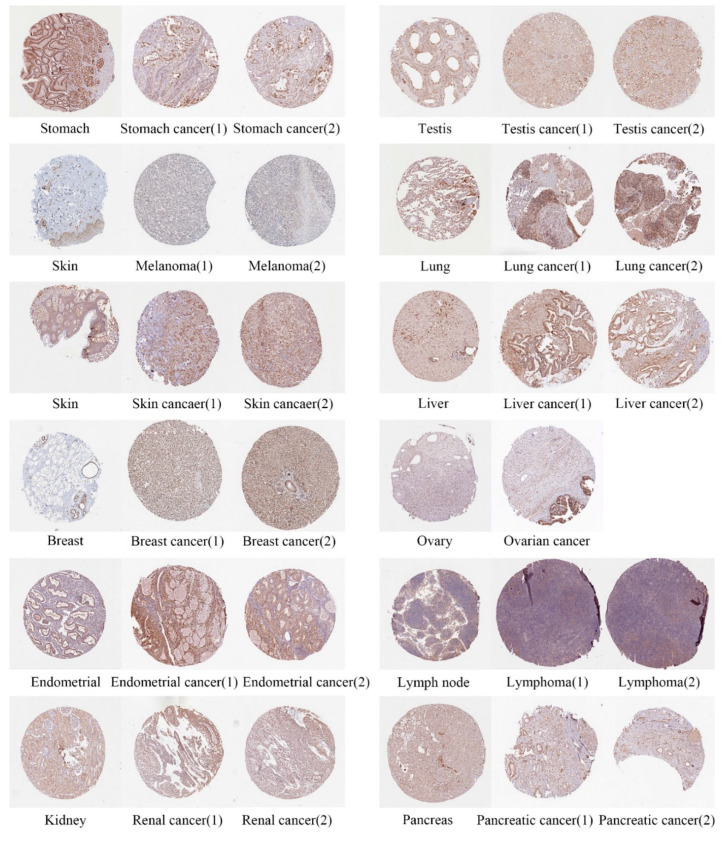
Immunohistochemical images of differential expressions of GNL3L protein in 12 tumors.

Next, the differences in GNL3L protein expression levels were analyzed using immunohistochemical images from the HPA database. Results from the HPA database showed that GNL3L protein was expressed in stomach cancer, testis cancer, melanoma, lung cancer, skin cancer, liver cancer, breast cancer, ovarian cancer, endometrial cancer, 10-lymphoma-1, 11-renal cancer-1, and 12-pancreatic cancer-1 significantly higher than in normal tissue (Figure 2).

### 3.2. GNL3L Expression Associated with Molecular Subtypes and Clinical Stages in Human Cancers

**Figure 3:**
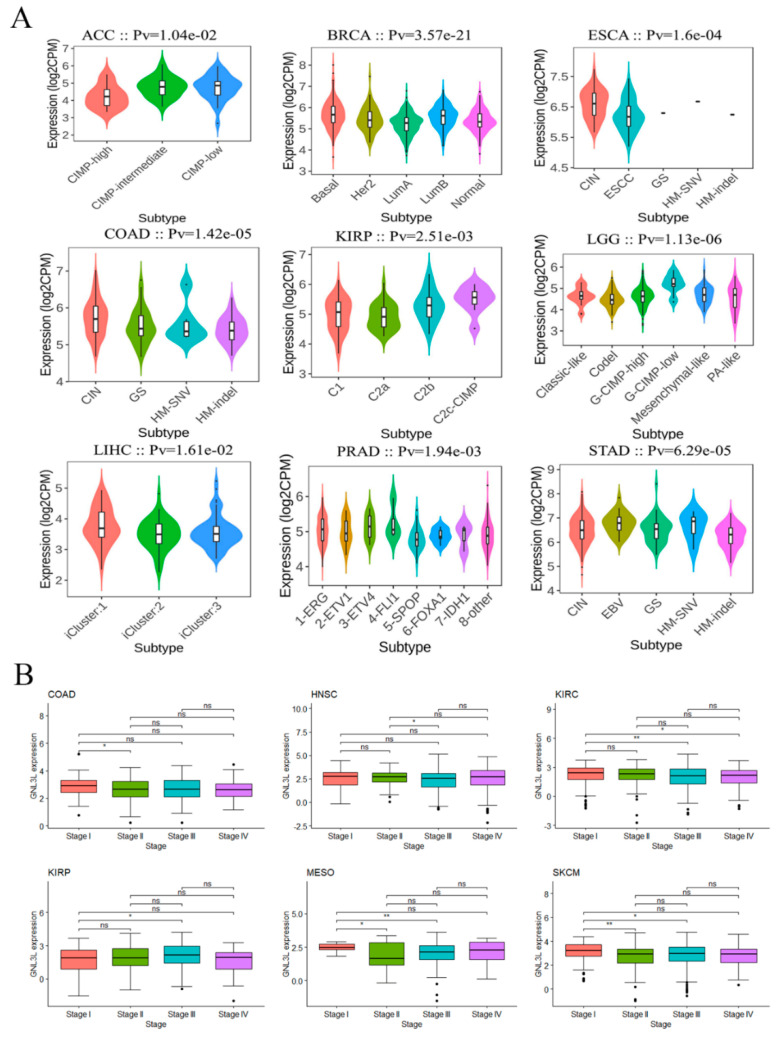
Correlation of GNL3L with cancer molecular subtypes and clinical stages. (**A**) GNL3L expression correlated with molecular subtypes in ACC, BRCA, ESCA, COAD, KIRP, LGG, LIHC, PRAD, and STAD. (**B**) Obvious discrepancy in GNL3L expression in dissimilar clinical stages in COAD, HNSC, KIRC, KIRP, MESO, and SKCM cancers. * *p* < 0.05; ** *p* < 0.01.

Moreover, the expression of GNL3L was investigated in several molecular subtypes and clinical phases. Using the TISIDB database, the expression of GNL3L was found to be significantly different among dissimilar molecular subtypes of ACC, BRCA, COAD, ESCA, KIRP, LGG, LIHC, PRAD, and STAD (Figure 3A). Then, the TCGA database GNL3L clinical data were analyzed, and the findings revealed that GNL3L expression varied depending on the clinical phase of COAD, HNSC, KIRC, KIRP, MESO, and SKCM (Figure 3B).

### 3.3. Genetic Variation and CNA Alterations of GNL3L in Human Cancer

**Figure 4:**
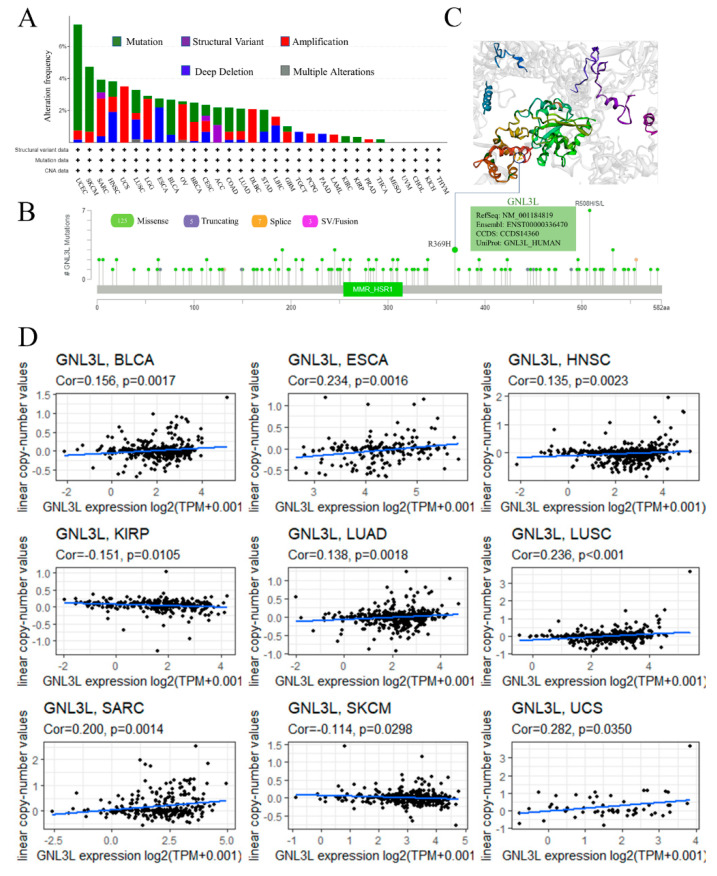
Genetic variation and mutation status of GNL3L. (**A**,**B**) show the mutation type and mutation frequency of GNL3L based on the cBioPortal database. (**C**) Mutation site R369H in the GNL3L 3D structure. (**D**) The correlation between GNL3L expression and the relative linear copy values of BLCA, ESCA, HNSC, LUAD, LUSC, SARC, UCS, KIRP, and SKCM was significant.

Cancer development and immunological tolerance are influenced by genetic and epigenetic alterations. GNL3L genetic variations and CNA changes were investigated further using the cBioPortal; GNL3L changes comprised mutations, amplifications, structural variations, deep deletions, and numerous alterations, according to the findings. Endometrial cancer, cutaneous melanoma, bladder cancer, and esophageal cancer all have mutations as the most prevalent form of change. GNL3L mutations were found in about 6.6% of UCEC patients; the “amplified” type of alterations accounted for most of the alterations in UCS (uterine carcinosarcoma) cases, with a frequency of about 3.51%; and the “deeply deleted” type of alterations accounted for most of the alterations in ESCA, with a frequency of about 2.2% (Figure 4A). The analysis showed that the main type of genetic variation in GNL3L was missense mutation. R369H site alteration was found in two COADs and one UCEC (Figure 4B). The missense mutation at the R369H site may lead to the abnormal structure and function of GNL3L protein in the body, which may cause disease. This mutation is harmful. The 3D structure of the GNL3L protein containing the R369H site is shown (Figure 4C).

Next, to explore the CNA situation of GNL3L, the correlation between GNL3L expression and the relative linear copy number was analyzed. The results of the research revealed that GNL3L expression is positively correlated with CNA in BLCA, ESCA, HNSC, LUAD, LUSC, SARC, and UCS and negatively correlated with CNA in KIRP and SKCM (Figure 4D). Thus, it was demonstrated that GNL3L expression correlates with relatively linear copy number values and affects CNA in a variety of cancers.

### 3.4. Prognostic Analysis of GNL3L Expression in Pan-Cancer

Univariate OS, PFI, and DSS analysis of data from 33 cancer types showed that GNL3L has different prognostic values in dissimilar types of cancer.

**Figure 5:**
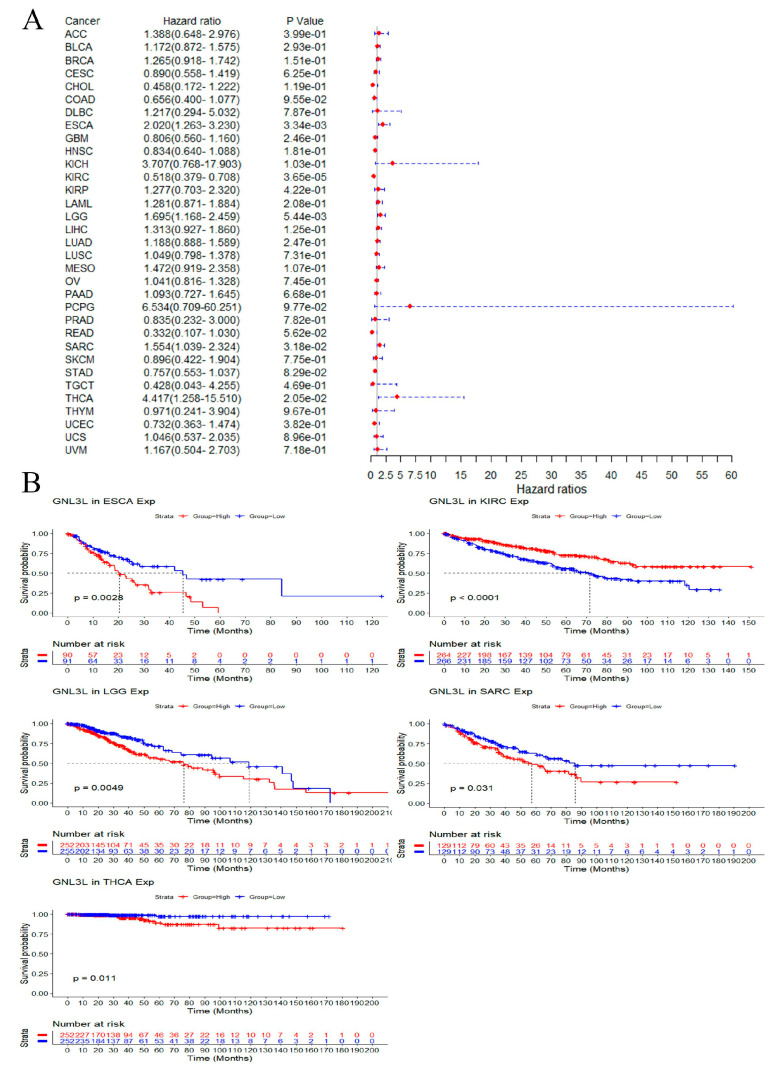
Correlation of GNL3L expression with OS in patients. (**A**) Forest plot of the OS risk ratio of GNL3L in 33 cancers. (**B**) Kaplan−Meier survival curves for patients with ESCA, KIRC, LGG, SARC, and THCA malignancies stratified by GNL3L expression levels.

In OS analysis, Cox proportional hazard model results showed that the high expression of GNL3L is a risk factor in ESCA (*p* = 3.34 × 10^−3^), LGG (*p* = 5.44 × 10^−3^), SARC (*p* = 3.18 × 10^−2^), and THCA (*p* = 2.05 × 10^−2^); meanwhile, high GNL3L expression is a protective factor in KIRC (*p* = 3.65 × 10^−5^) (Figure 5A). In addition, Kaplan–Meier OS curves showed that high GNL3L expression in ESCA (*p* = 0.0028, HR = 2.020), LGG (*p* = 0.0049, HR = 1.695), SARC (*p* < 0.0001, HR = 1.554), and THCA (*p* = 0.0011, HR = 4.417) with an OS prognosis is poorer. The low expression of GNL3L was interrelated with a poorer OS prognosis in KIRC (*p* = 0.0015, HR = 0.581) (Figure 5B).

**Figure 6:**
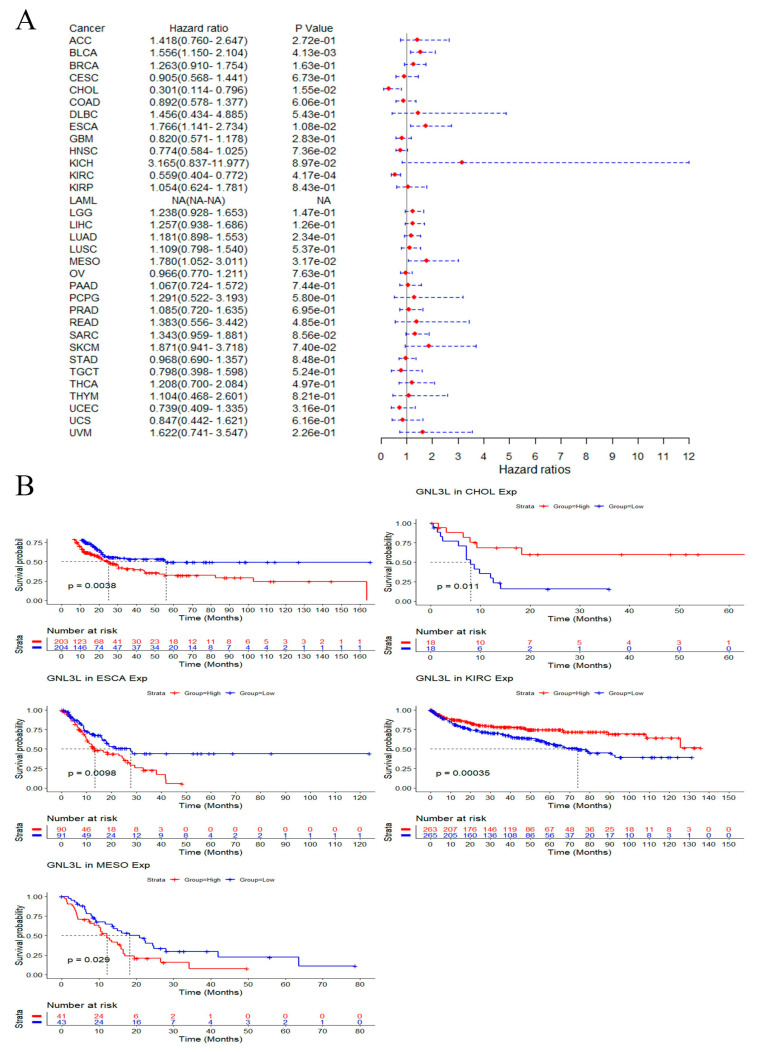
Correlation of GNL3L expression with PFI in patients. (**A**) Forest plot of PFI risk ratios for GNL3L in 33 cancers. (**B**) Kaplan–Meier PFI curves for patients in BLCA, CHOL, ESCA, KIRC, and MESO malignancies stratified by GNL3L expression levels.

In PFI analysis, the Cox proportional hazard model results showed that high GNL3L expression is a risk factor for ESCA (*p* = 1.08 × 10^−2^), BLCA (*p* = 4.13 × 10^−3^), and MESO (*p* = 3.17 × 10^−2^); meanwhile, high GNL3L expression is a protective factor in CHOL (*p* = 1.55 × 10^−2^), and KIRC (*p* = 4.17 × 10^−4^) (Figure 6A). In addition, Kaplan–Meier PFI curves showed a high GNL3L expression in BLCA (*p* = 0.0038, HR = 1.556), ESCA (*p* = 0.0098, HR = 1.766), and MESO (*p* = 0.029, HR = 1.780) with a poorer PFI prognosis. Low GNL3L expression in CHOL (*p* = 0.011, HR = 0.301) and KIRC (*p* = 0.00035, HR = 0.559) had a poorer prognosis for PFI (Figure 6B).

**Figure 7:**
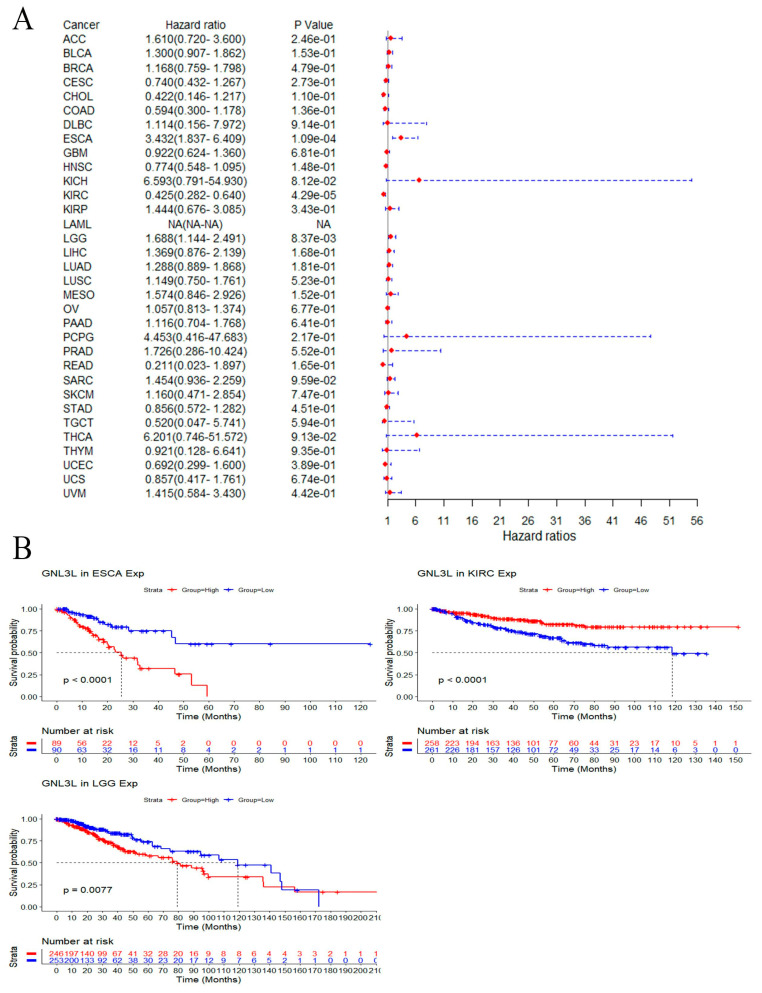
Correlation of GNL3L expression with DSS in patients. (**A**) Forest plot of DSS risk ratios for GNL3L in 33 cancers. (**B**) Kaplan–Meier DSS curves for patients stratified by different GNL3L expression levels in ESCA, KIRC, and LGG cancers.

In DSS analysis, the Cox proportional hazards model results showed that high GNL3L expression is a risk factor in ESCA (*p* = 1.08 × 10^−2^) and LGG (*p* = 8.37 × 10^−3^); meanwhile, high GNL3L expression is a protective factor in KIRC (*p* = 4.29 × 10^−5^) (Figure 7A). In addition, Kaplan–Meier DSS curves showed high GNL3L expression in ESCA (*p* < 0.0001, HR = 3.432) and LGG (*p* = 0.0077, HR = 1.688) with poorer DSS prognosis. Low GNL3L expression in KIRC (*p* < 0.0001, HR = 0.425) had a poorer prognosis for DSS (Figure 7B).

### 3.5. Correlation of GNL3L with TMB, MSI, and MMR

**Figure 8:**
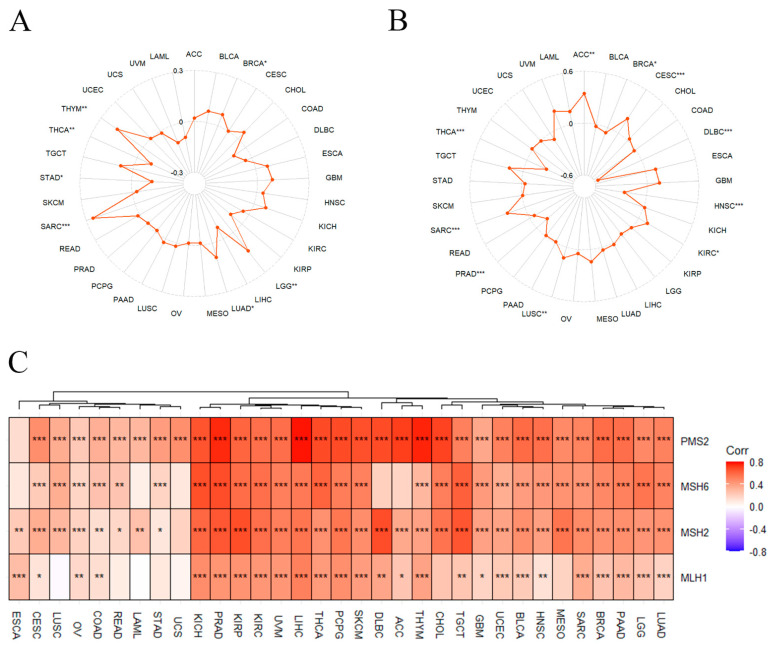
GNL3L expression correlated with TMB, MSI, and MMR. (**A**) Radar plot of the correlation between GNL3L expression and TMB. (**B**) Radar plot of the correlation between GNL3L expression and MSI. (**C**) Heatmap of the correlation between GNL3L expression and MMR genes. * *p* < 0.05; ** *p* < 0.01 and *** *p* < 0.001.

After determining the prognostic value of GNL3L, the association between GNL3L and TMB, MSI, and MMR in 33 cancers was discussed. A TMB and MSI correlation radar plot showed that GNL3L is associated with TMB in BRCA, LGG, LUAD, SARC, STAD, THCA, and THYM, and in ACC, BRCA, CESC, DLBC, HNSC, KIRC, LUSC, PRAD, SARC, and THCA, it is related to MSI (Figure 8A,B). The heatmap of the correlation between GNL3L and MMR showed that GNL3L is co-expressed and significantly associated with PMS2 in 32 cancers except for ESCA; GNL3L is associated with MSH6 in 28 cancers except for ESCA, LAML, UCS, DLBC, and ACC, where it is co-expressed and significantly correlated; GNL3L was co-expressed with MSH2 in 32 cancers except for UCS; GNL3L was co-expressed with MLH1 in 26 cancers except for LUSC, READ, LAML, STAD, UCS, CHOL, and MESO, which are significantly correlated (Figure 8C).

### 3.6. Correlation between GNL3L and Immune Microenvironment

**Figure 9:**
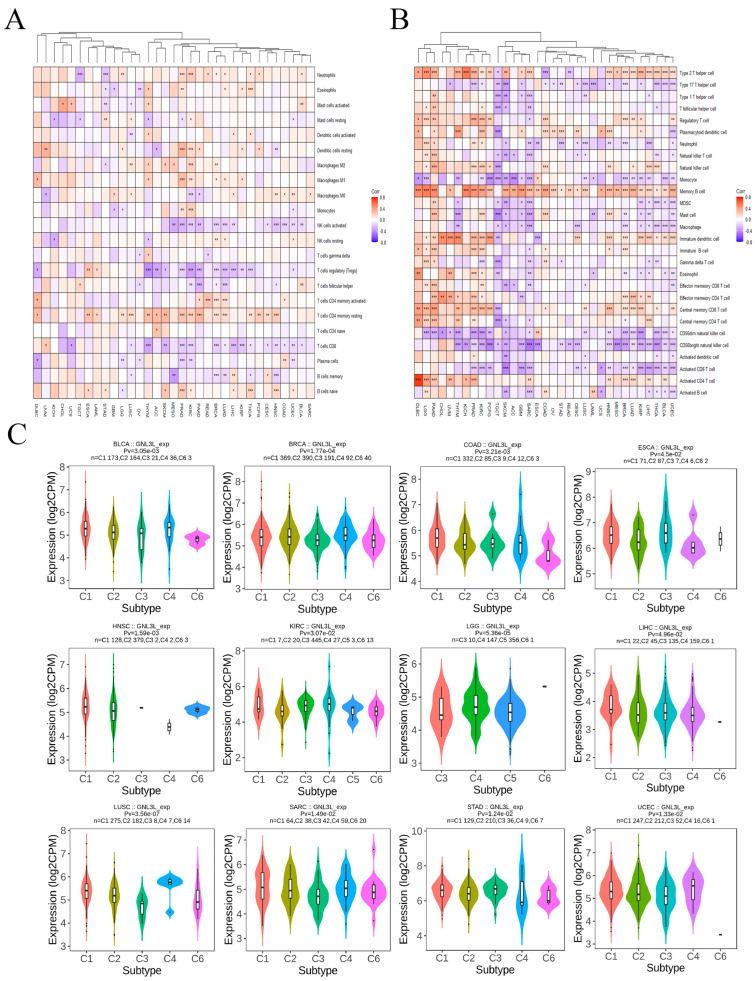
Correlation of GNL3L expression with tumor-infiltrating immune cells. (**A**) Heatmap of GNL3L expression correlation with 22 immune cells based on CIBERSORT algorithm. (**B**) Heatmap of correlation between GNL3L expression and 27 immune cells based on ssGSEA algorithm. (**C**) GNL3L was differentially expressed in different immune subtypes. * *p* < 0.05; ** *p* < 0.01 and *** *p* < 0.001.

Following the discovery of GNL3L’s predictive usefulness, the researchers investigated the link between GNL3L and tumor-infiltrating immune cells in 33 malignancies. The CIBERSORT method was used to assess the components of the tumor immune cell microenvironment in 33 malignancies from the TCGA. Clustered heatmaps based on the correlation between GNL3L and immune cells showed that GNL3L is positively correlated with T cell CD4 memory, part of the majority of cancers, especially ESCA (Spearman r = 0.26, *p* = 0.001), PAAD (Spearman r = 0.31, *p* = 3.75 × 10^−5^), and DLBC (Spearman r = 0.30, *p* = 0.043). GNL3L is negatively correlated with T cell regulation (Tregs) in the majority of cancers; however, a positive correlation was found in ESCA (Spearman r = 0.25, *p* = 0.001) and LAML (Spearman r = 0.17, *p* = 0.043). GNL3L was shown to be negatively correlated with T cell CD8 in the majority of cancers; however, it is positively correlated in UVM (Spearman r = 0.23, *p* = 0.045) (Figure 9A).

Furthermore, the components of the tumor immune cell microenvironments of 33 cancers from TCGA were evaluated with the ssGSEA algorithm again. The clustered heatmap based on the correlation between GNL3L and immune cells revealed that GNL3L is positively correlated with Memory B cells in the majority of cancers, especially KICH (Spearman r = 0.503, *p* = 1.95 × 10^−5^), GBM (Spearman r = 0.418, *p* = 1.81 × 10^−7^), PAAD (Spearman r = 0.470, *p* = 4.28 × 10^−11^), and ACC (Spearman r = 0.345, *p* = 0.0018). This also showed that GNL3L is negatively correlated with CD56 bright natural killer cells in the majority of cancers, especially MESO (Spearman r = −0.394, *p* = 0.00027), SARC (Spearman r = −0.476, *p* = 4.44 × 10^−16^), and ESCA (Spearman r = −0.380, *p* = 1.32 × 10^−6^). GNL3L is negatively correlated with the CD56 dim natural killer cell in the majority of cancers; however, a positive correlation was discovered in ESCA (Spearman r = 0.212, *p* = 0.0087). We also found that GNL3L is positively correlated with Type 2 T helper cells in the majority of cancers; however, it is negatively correlated in TGCT (Spearman r = −0.182, *p* = 0.026), COAD (Spearman r = −0.304, *p* = 3.45 × 10^−11^), and READ (Spearman r = −0.21, *p* = 0.0075) (Figure 9B).

Then, the analysis results of the CIBERSORT algorithm and ssGSEA algorithm were combined for further analysis. The analysis results of both algorithms showed that GNL3L is positively correlated with eosinophils (CIBERSORT: r = 0.128, *p* = 0.0050; ssGSEA: r = 0.192, *p* = 2.21 × 10^−5^) in PRAD. GNL3L is positively correlated with neutrophils in KIRC (CIBERSORT: r = 0.211, *p* = 1.02 × 10^−6^; ssGSEA: r = 0.112, *p* = 0.0103), and GNL3L is positively correlated with B cell memory in LIHC (CIBERSORT: r = 0.139, *p* = 0.0075; ssGSEA: r = 0.173, *p* = 0.0009).

The TISIDB database was used to investigate the influence of GNL3L expression on distinct immune subtypes in human malignancies. Immune subtypes are divided into six types, including C1 (wound healing), C2 (IFN-γ dominant), C3 (inflammatory), C4 (lymphocyte depletion), C5 (immune quiet), and C6 (TGF-b dominant). The results showed that GNL3L expression is significantly different from different the immune subtypes of BLCA, BRCA, COAD, ESCA, HNSC, KIRC, LGG, LIHC, LUSC, SARC, STAD, and UCEC (Figure 9C).

### 3.7. GNL3L-Related Gene Enrichment Analysis

**Figure 10:**
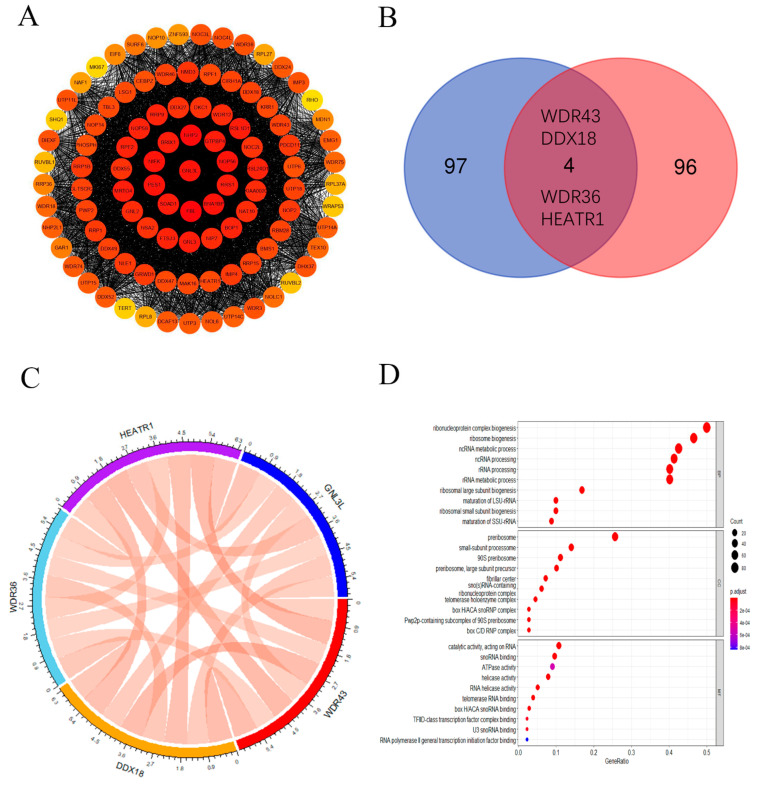
GNL3L-related gene enrichment analysis. (**A**) Co-expression network of GNL3L genes displayed in Cytoscape software. (**B**) Venn diagram of the genome based on the STRING database and the GEPIA2 database. (**C**) GNL3L, WDR43, DDX18, WDR36, and HEATR1 gene expression correlation chord plots. (**D**) Plot of the results of the GO enrichment analysis of GNL3L and its related genes.

In order to further study the molecular mechanism of GNL3L in carcinogenesis, we first obtained the top 100 proteins related to GNL3L using the STRING tool, constructed a PPI network, and expressed the network of these gene interactions in the Cytoscape software (Figure 10A). Subsequently, the GEPIA2 database was used to obtain the top 100 genes associated with GNL3L expression. Then, the genes corresponding to the 100 proteins in the PPI and the top 100 genes obtained in the GEPIA2 database were intersected, and a Venn diagram was drawn to obtain the four most common genes in the above two datasets: WDR43, DDX18, WDR36, and HEATR1 (Figure 10B). Then, a gene expression correlation analysis between GNL3L and the above four genes was performed, and a chord diagram was drawn (Figure 10C). In it, the line in the figure represents the correlation information between two genes; red represents positive correlation; green represents negative correlation; and the thicker the line, the higher the correlation strength. The correlation coefficient can be calculated with the disc scale. It can be seen from the figure that the correlation coefficients between the five genes are all between 0.7 and 0.85, and the correlation is high. To further investigate the function and pathway enrichment analysis of GNL3L, KEGG and GO enrichment analyses were performed using the 197 genes above. The KEGG pathway analysis showed that GNL3L is associated with the ribosome pathway in eukaryotes. In addition, GO analysis showed that most of these genes were associated with ribonucleoprotein, rRNA processing, rRNA metabolic process, and ncRNA metabolic processes in the BP class; pre-ribosomal, telomerase holoenzyme complex, and small subunit process groups in the CC class; and catalytic activity, helicase activity, and ATPase activity acting on RNA in the MF class (Figure 10D). To further explore the association between GO analysis results and the cellular localization and functions of GNL3L, the cellular localization and functions of GNL3L gene were analyzed using the GeneCards database. The GeneCards database showed that GNL3L is subcellularly localized to the nucleus and nucleolus, and it is essential for ribosomal pre-rRNA processing and cell proliferation. Through GO analysis results and the cellular localization and functions of GNL3L, it can be seen that GNL3L may affect the occurrence of cancer through ribosomal RNA processing.

### 3.8. Establishment of ESCA Prediction Model

**Figure 11:**
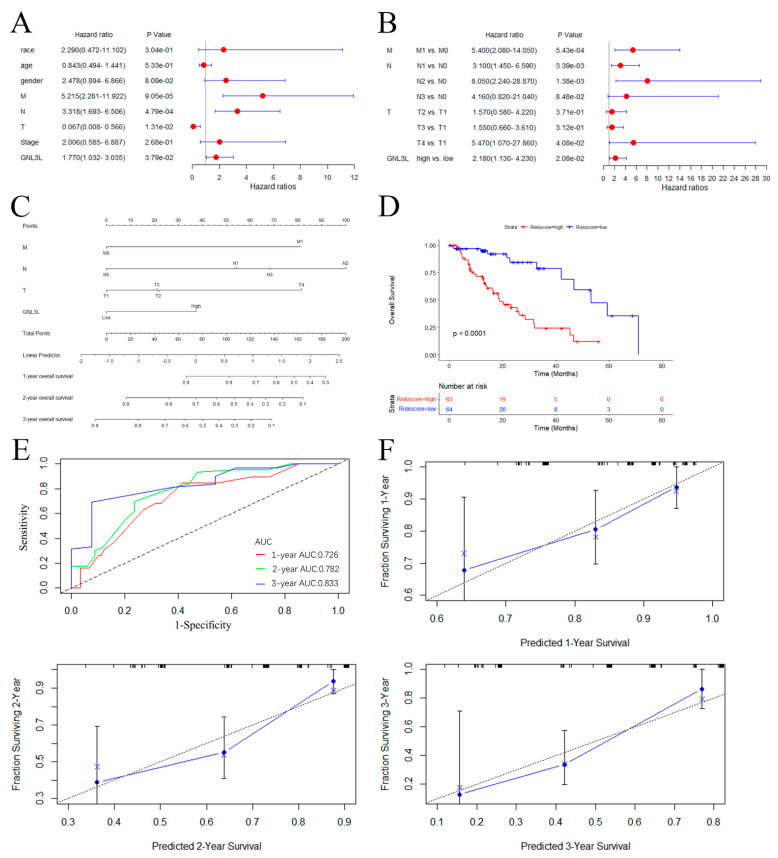
ESCA prediction model building and evaluation. (**A**) Forest plot of univariate Cox regression results. (**B**) Forest plot of multi-factor Cox regression results. (**C**) The nomogram based on GNL3L and M, T, and N stages. (**D**) Kaplan–Meier curves plotted after stratifying high−risk and low−risk patients based on median risk scores derived from the nomogram. (**E**) Evaluation of the ROC curve of the nomogram. (**F**) Evaluation of the calibration curve for the nomogram.

In all the above analyses, the results indicated that GNL3L expression is significantly correlated with prognosis and immune cell infiltration in various cancers. Especially in ESCA, GNL3L showed high expression, poor prognosis, and significant correlation with molecular subtype, immune subtype, immune cell, OS, PFI, and DSS curves. Therefore, to further investigate GNL3L and understand the prognostic role of GNL3L in ESCA, we tested whether the GNL3L gene is an independent prognostic factor using univariate and multivariate Cox regression analysis. Univariate Cox regression analysis revealed that M (*p* < 0.001), N (*p* < 0.001), T (*p* = 0.013), and the GNL3L gene (*p* = 0.038) are correlates of the survival prognosis in ESCA patients (Figure 11A). A multivariate COX regression analysis using only the statistically significant features of the univariate COX model followed. The results showed that M (M1 vs. M0 *p* < 0.001), N (N1 vs. N0 *p* = 0.003, N2 vs. N0 *p* = 0.001), T (T4 vs. T1 *p* = 0.041), and the GNL3L gene (high vs. low *p* = 0.021) are all independent risk factors for predicting OS dysplasia in ESCA patients (Figure 11B). Among them, T staging indicates the size of the primary tumor state; N staging indicates the status of regional lymph node metastasis; and M staging indicates the presence or absence of distant metastasis. The results validated and confirmed that the GNL3L gene can be used as an independent prognostic factor for ESCA patients.

A nomogram based on the results of the multivariate Cox analysis for predicting the probability of survival in ESCA patients was then constructed (Figure 11C) to further improve GNL3L prediction accuracy. To assess the predictive risk potential of this nomogram, a Kaplan–Meier analysis was performed by stratifying all patients based on the median risk score sourced from the nomogram, consisting of M0, N1, and T3 staging and GNL3L gene expression levels (Figure 11D), with high-risk patients exhibiting remarkably disadvantaged survival conclusions in terms of OS rates in contrast to low-risk patients. To validate the prediction model, ROC curves and calibration graphs were drawn to evaluate the prediction performance. The results showed AUC values of 0.726, 0.782, and 0.833 for the ROC curves at 1 year, 2 years, and 3 years, respectively (Figure 11E). The calibration curve showed good agreement between the prediction of the line chart and the actual observation (Figure 11F). These results further strengthen the clinical significance of GNL3L expression in relation to M0, N1, and T3 staging, indicating that the model has a good predictive power in predicting survival outcomes in ESCA patients.

### 3.9. Analysis of Clone Formation Assays of GNL3L Overexpression and Knockdown

**Figure 12:**
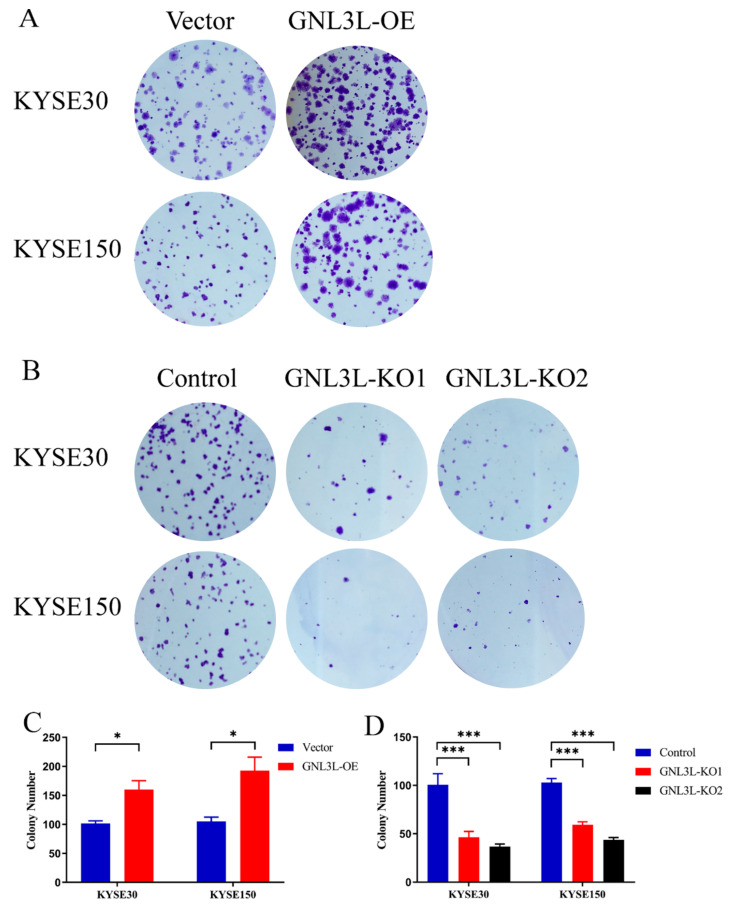
Results of clone formation assays for GNL3L overexpression and knockdown. (**A**,**C**) Graph of the results of clone formation assays with GNL3L overexpression. (**B**,**D**) Graph of the results of clone formation assays for GNL3L knockdown. * *p* < 0.05; *** *p* < 0.001.

By means of transient transfection technology, we increased or decreased the expression of the GNL3L gene in two esophageal cancer cells (KYSE30 and KYSE150) and performed clone formation assays, respectively. In the GNL3L overexpression experiment, the number of cell clones in the KYSE30-Vector (control group), KYSE30-GNL3L-OE, KYSE150-Vector, and KYSE150-GNL3L-OE groups was 101.667 ± 7.638, 160 ± 26.458, 105 ± 13.229, and 192.667± 40.266. From the statistical results, the number of clones in the overexpression group of the two cell lines was significantly higher than in the control group (KYSE30, t = 3.669, *p* = 0.0214; KYSE150, t = 3.583, *p* = 0.0231), which was statistically significant (Figure 12A,C). In the GNL3L knockdown experiment, two sequences, KO1 and KO2, were designed to knockdown GNL3L. The number of clones in the GNL3L-KO2 group was 100.667 ± 19.629, 46.333 ± 10.599, 36.667 ± 4.726, 103 ± 7, 59.333 ± 5.132, 43.667 ± 4.041. From the statistical results, the number of clones in the knockdown group of the two cell lines was significantly reduced compared to the control group (*p* = 0.002054, *p* = 0.000031); it was statistically significant (Figure 12B,D).

The results of two clone formation assays showed that the clone formation ability of esophageal cancer cells was significantly increased after the high expression of GNL3L, and the clone formation ability of esophageal cancer cells was significantly reduced after the low expression of GNL3L. Furthermore, it can be seen that GNL3L significantly affects the proliferation ability of esophageal cancer cells.

## 4. Discussion

Previous studies have shown that green fluorescent protein (GFP) can be distributed within the nucleolus without changing the conformation and function of the original protein when attached to the nucleolus internal localization signal (NoLS) protein [33]. Rao et al. modified the ends of GNL3L with GFP and observed the organelle localization of GNL3L-GFP in Cos-7 cells via fluorescence microscopy and found that it was mainly localized in the nucleolus and partially distributed in the nucleoplasm; the same results were obtained in U2OS cells [34]. Therefore, GNL3L is mainly distributed in the nucleolus and partially in the nucleoplasm. Some later studies have shown that GNL3L is a nucleoplasmic shuttle protein that can shuttle between the nucleolus, nucleus, and cytoplasm. Other researchers found that GNL3L plays an important role in a variety of cancers and plays an important role in cell proliferation and ribosome synthesis [35,36,37]. However, we did not find any literature on the pan-cancer analysis of GNL3L. In this context, we analyzed the prognostic and immunological value of GNL3L in pancytopenia.

In this study, we performed multi-omics analyses of the genome, transcriptome, proteome, and epigenome. Firstly, the expression level of GNL3L gene was studied with TIMER2 and GEPIA. The results showed that GNL3L is highly expressed in BLCA, BRCA, CHOL, COAD, ESCA, GBM, HNSC, LIHC, LUAD, LUSC, READ, STAD, UCEC, DLBC, LAML, LGG, and TGCT. However, GNL3L is lowly expressed in KIHC, KIRC, KIRP, SKCM, and THCA. Then, GNL3L protein expression levels were investigated using the UALCAN database and HPA database, and immunohistochemical images in various cancers were studied. This indicates that GNL3L is differentially expressed in the majority of cancers and is a potential biomarker. The relationship between GNL3L expression and molecular subtypes and clinical stages in cancer was discussed next, leading to a study of the potential mechanisms of action. The results suggested that GNL3L is associated with both molecular subtypes and clinical stages in most cancers and may play a role in cancer growth and progression. Some studies have shown that the vast majority of cancers are caused by genetic mutations that alter cancer cells and enhance their ability to fight surrounding normal cells, which is also the focus of research on carcinogenesis [38,39]. In the current study, the genetic variation and CNA variation of GNL3L were further explored using cBioPortal. The results indicated that GNL3L is genetically altered in a variety of cancers, and the types of variation are mainly mutations, structural variants, and deep deletions. In the present study, the relevance of GNL3L expression in the prognostic value of cancer was also analyzed. Univariate OS, PFI, and DSS analysis of 33 cancer types showed that GNL3L has different prognostic values in different cancer types.

In recent years, with the rise of immune checkpoint inhibitors, traditional tumor treatment strategies have greatly changed. In theory, the greater the number of tumor mutations, the more likely it is to generate neoantigens [40]. Therefore, TMB is an important indicator for predicting immune efficacy, and MSI is also an important immune examination marker. The results of this study show that the expression of GNL3L is associated with TMB and MSI in various cancers, so the high expression of GNL3L may affect the treatment of immune checkpoint inhibitors. At present, the common immunohistochemical method is to detect the expression of the MMR genes MLH1, MSH2, MSH6, and PMS2 in the tumor group [41]. If the result shows that any protein is completely missing, it is interpreted as dMMR. Therefore, to continue the discussion on the correlation of GNL3L expression with immunotherapy, we analyzed the correlation of GNL3L with these four genes in 33 cancers. The heatmap results showed that GNL3L was only positively correlated with PMS2 in UCS, which co-expressed and significantly correlated with at least two of these genes in the remaining cancers. Therefore, we reasoned that GNL3L could be a novel biomarker associated with immune checkpoint inhibitors.

The tumor immune microenvironment has recently become a prominent issue in tumor research [42]. In addition, the effect of GNL3L on the immune microenvironment is rarely studied. The link between GNL3L and tumor-infiltrating immune cells was studied in 33 malignancies in this study. The results of the CIBERSORT algorithm and the ssGSEA algorithm showed that GNL3L is significantly associated with immune cells such as the Memory B cell, CD56 bright natural killer cell, CD56 dim natural killer cell, and Type 2 T helper cell in the majority of cancers. In most cancer types, there are remarkable dissimilarities in GNL3L expressions in different immune subtypes, which may demonstrate that GNL3L is a novel immune-related biomarker. These new findings represent a significant advance in defining the major role of GNL3L in the immune microenvironment and cancer analysis. 

To further investigate the molecular mechanism of GNL3L in carcinogenesis, a PPI network was constructed to enrich the function and pathway analysis of GNL3L. A KEGG pathway analysis indicated that GNL3L has an influence on the ribosomal pathway of ribosome genesis in eukaryotes. Moreover, GO analysis showed that GNL3L genes are associated with ribonucleoproteins, rRNA processing, metabolic processes, and ncRNA metabolic processes in the BP class; preribosome, telomerase holoenzyme complex, and small-subunit processome in the CC class; and MF class catalytic activity acting on RNA, helicase activity, and ATPase activity. A gene enrichment analysis showed that GNL3L may be closely related to metabolic processes and ribosome synthesis.

Meanwhile, in this study, we found that GNL3L expression is significantly correlated with the prognosis and immune cell infiltration of various cancers, such as ESCA, KIRC, LGG, and SARC. The high expression of GN3L, as well as significant differences in molecular subtypes and immune subtypes in OS, PFI, and DSS analyses, are associated with a shorter survival time in ESCA patients compared to ESCA patients with low GNL3L expressions, and GNL3L is significantly associated with most immune cells in ESCA patients. In addition, studies have shown that GNL3L may be a potential prognostic marker for ESCA [8,37]. To more deeply analyze the prognostic worth and clinical value of GNL3L in cancer, finally, a predictive model of ESCA was constructed. A multivariate Cox-based risk line plot for predicting the probability of survival in ESCA patients was constructed by placing GNL3L with statistically significant clinical characteristics in a univariate Cox analysis, and the ROC curves indicated that GNL3L was highly predictable. For example, patients at the M0, N1, and T3 stages and with higher GNL3L gene expressions would receive a total of approximately 110 points, with predicted OS rates of approximately 76.0%, 47.0%, and 27.0% at 1 year, 2 years, and 3 years, respectively. These results indicate that the model has a good predictive ability and has some clinical value. Finally, in order to further study the effect of GNL3L expression on esophageal cancer, we supplemented a biological experiment and performed a clone formation assay of GNL3L. From the results of overexpression and knockdown experiments, it can be seen that GNL3L significantly affects the proliferation ability of esophageal cancer cells.

In conclusion, the results of the GNL3L expression analysis showed that the GNL3L gene is differentially expressed in the vast majority of cancers and correlated with molecular subtypes and clinical stages. GNL3L protein expression is significantly higher in various cancers than in normal tissues. Later, we found that GNL3L is a missense mutation in most cancers. Then, the analysis results of OS, PFI, and DSS showed that GNL3L has different prognostic values in different cancers. Next, in immunoassays, GNL3L was found to be significantly associated with TMB, MSI, MMR, and various immune cells in various cancers. Meanwhile, in order to further analyze the prognostic value and clinical value of GNL3L in cancer, we constructed a multivariate Cox-based risk nomogram for predicting the survival probability of ESCA patients, and we found that the model has a good predictive ability. Finally, we supplemented a biological experiment to perform a clone formation experiment on GNL3L. From the results of the overexpression and knockdown experiments, it can be seen that GNL3L significantly affects the proliferation ability of esophageal cancer cells. The results of these analyses illustrate the expression, prognosis, and clinical value of GNL3L, demonstrating the importance of GNL3L expression in the early detection and prognosis of multiple cancers. Therefore, GNL3L can serve as a potential pan-cancer biomarker.

## 5. Conclusions

Although we integrated multiple databases and performed a comprehensive and systematic analysis of GNL3L, this study still has some limitations. First, public databases were used, and the quality of data collection and the procedures employed to obtain the data may differ from one source to the next, affecting the conclusions of some analyses. Second, this study only studied data analyses of GNL3L and various cancers and only performed a biological experiment with a clone formation assay. More biological experimental work is needed to determine the precise function of GNL3L in cancer occurrence and prognosis.

In conclusion, we analyzed the expression characteristics, enrichment value, and prognostic value of GNL3L in pan-cancer and established an ESCA prediction model. At the same time, we performed clone formation assays to further verify the expression, prognosis, and clinical value of GNL3L. These results demonstrate the importance of GNL3L expression in the discovery and prognosis of cancer. Therefore, GNL3L can be used as a potential prognostic immune biomarker.

## Data Availability

Not applicable.
